# Supplementary material for: CryoTEN: Efficiently Enhancing Cryo-EM Density Maps Using Transformers
Source: bioRxiv. 2024 Sep 11:2024.09.06.611715. Preprint. [Version 1] doi: 10.1101/2024.09.06.611715 (PMC11418965; doi:10.1101/2024.09.06.611715)
Supplement: Supplement 1 [file media-1.pdf]

CryoTEN: Efficiently Enhancing Cryo-EM Density Maps using Transformers - Supplementary Information

Joel Selvaraj[1,2], Liguo Wang[3], Jianlin Cheng[1,2,*]

1. Department of Electrical Engineering and Computer Science, University of Missouri, Columbia - 65211, MO, United States.
2. NextGen Precision Health, University of Missouri, Columbia - 65211, MO, United States.
3. Laboratory for BioMolecular Structure (LBMS), Brookhaven National Laboratory, Upton - 11973, NY, United States.

* - corresponding author (chengji@missouri.edu)

Supplementary Figure 1: Plots of map-model validation metrics computed on deposited cryo-EM half maps and CryoTEN processed half maps

Computed various map validation metrics on deposited cryo-EM half maps (one of the half map pairs is used) and CryoTEN processed maps in our test set containing 70 half map pairs. Half violin and box plots of **(a)** unmasked FSC@0.143 resolution, **(b)** unmasked FSC@0.5 resolution (2 outliers with FSC@0.5 > 20Å in deposited half maps are hidden for better visualization), **(c)** average Q-score, **(d,e,f)** CC_box, CC_mask and CC_peaks scores respectively.

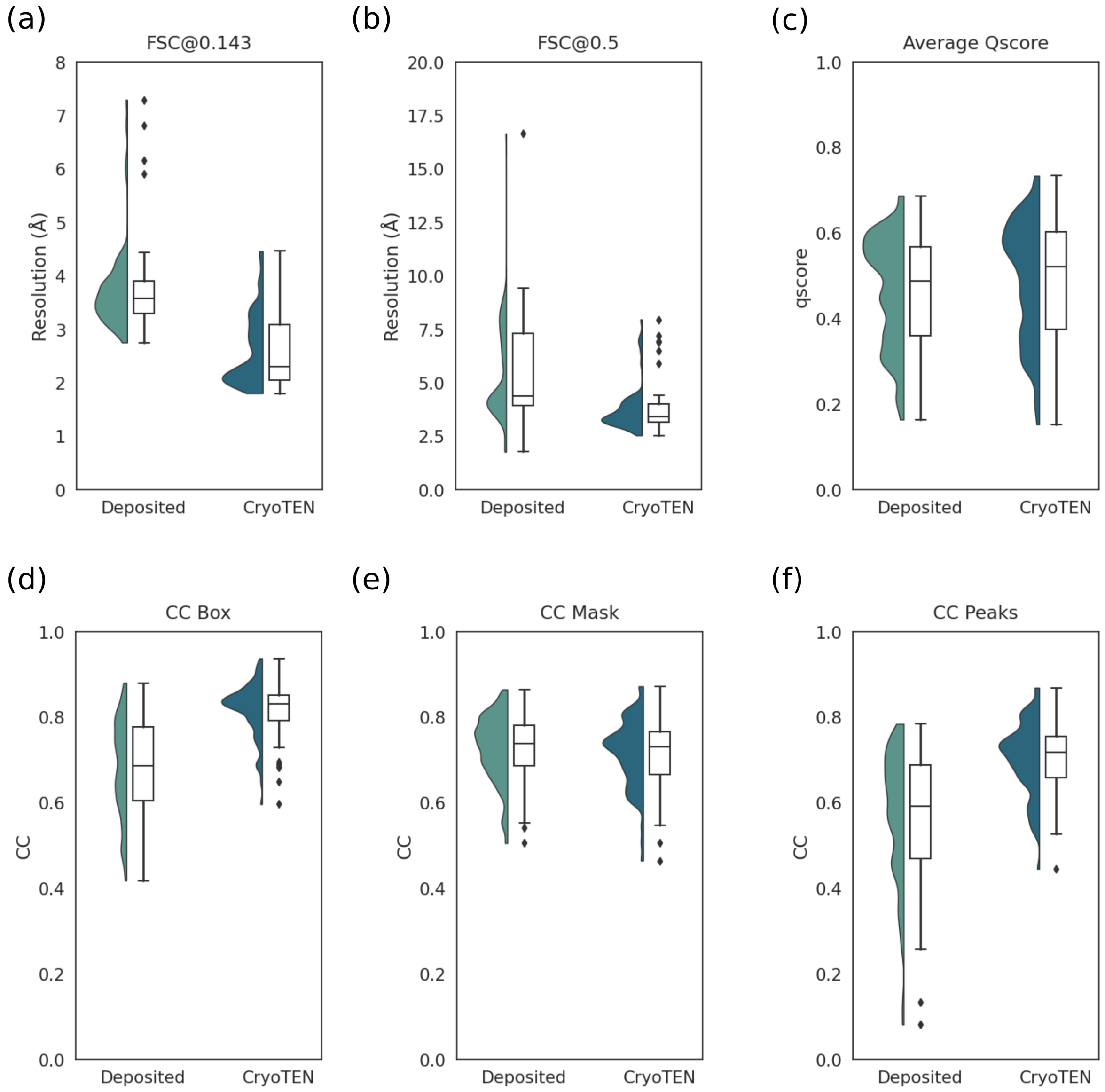

Supplementary Figure 2: An example showing improvement in CryoTEN processed maps

An example that shows improvement in map quality when using CryoTEN. (a) and (b) compares the deposited density map (blue) and CryoTEN enhanced map (green) of EMD-22937 at various contour levels respectively. Each map overlapped with the corresponding PDB structure (PDB ID: 7KNA) (red). Since the density value distribution varies between maps, we match the density volume between the deposited and CryoTEN processed maps to make a fair comparison. The dotted circles in the images of the maps highlight the regions where CryoTEN substantially removes noise and/or adds more structural detail.

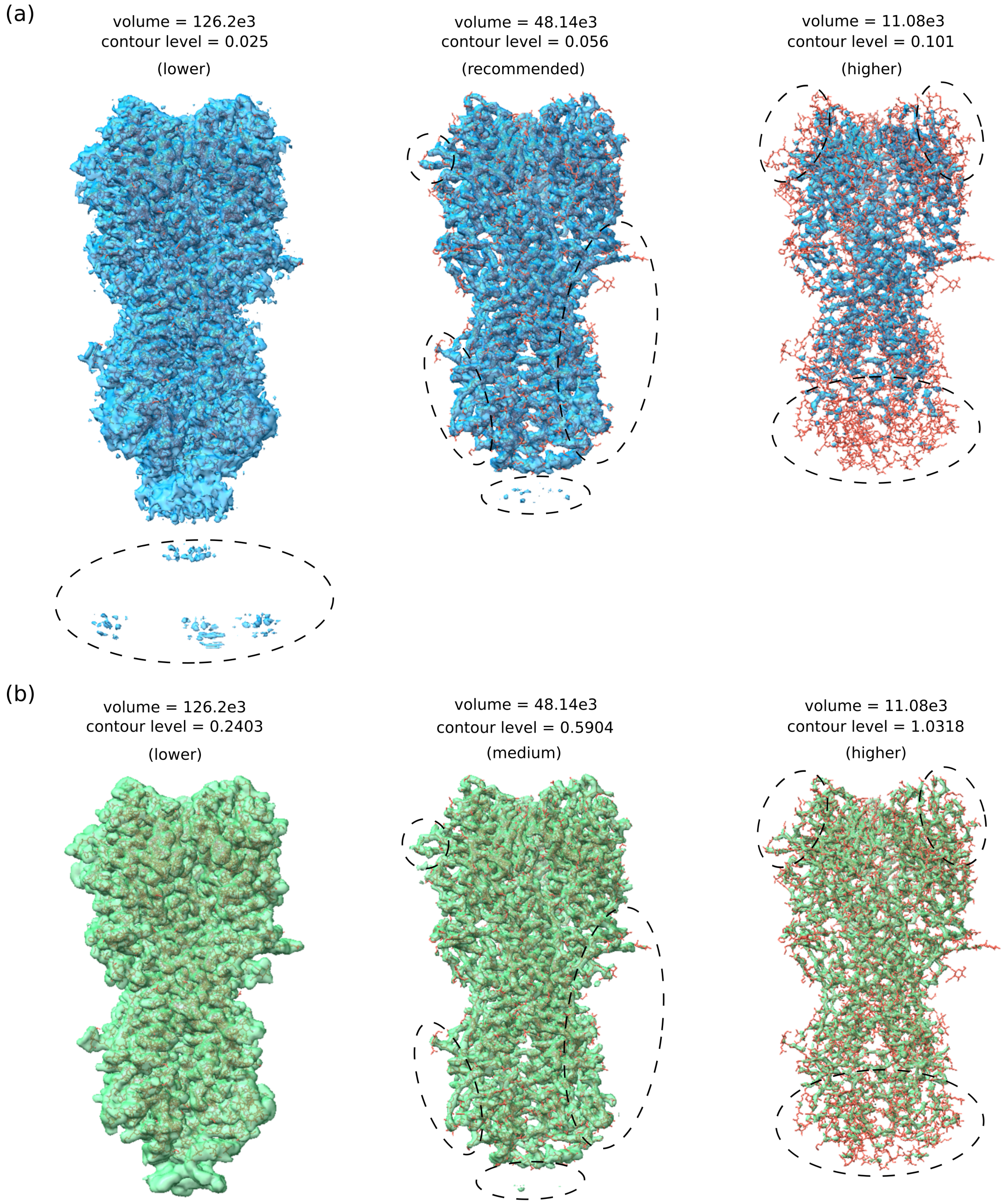

Supplementary Table 1: Runtime Benchmark of CryoTEN, DeepEMHancer and EM-Ready on 20 maps

We ran CryoTEN, DeepEMhancer, and EMReady models on 20 maps in the test set with a batch size of 40. The time taken to process each map by these models is tabulated below. From the total time taken to process 20 maps, we can observe that CryoTEN performed significantly faster than DeepEMhancer and EMReady.

EMDB ID	CryoTEN Time Taken (mins)	DeepEMhancer Time Taken (mins)	EMReady Time Taken (mins)
35363	2.010357	54.836444	14.353148
26831	1.214203	37.408689	1.209833
27630	1.569806	38.069682	32.110613
23092	1.492781	48.146252	8.832931
13867	1.193822	41.560315	30.045555
23970	1.173997	38.321360	20.812409
26978	2.143782	37.432961	5.202853
22884	1.896650	54.818219	2.000178
32765	2.644996	47.408696	61.382500
24784	1.632092	42.320093	31.831395
32328	1.292059	40.041838	2.611483
4032	2.155701	70.056347	22.552169
26806	1.557495	45.533855	6.369265
20226	1.344403	35.729695	5.676419
33242	1.560156	34.966081	36.235096
14847	1.303207	47.767493	7.853368
34369	1.601426	35.538966	25.820971
10800	2.093365	50.292193	16.810930
15531	1.230027	28.490879	2.447058
32940	2.177127	36.571903	58.840337
**Total (mins)**	33.29	865.31	393.0
